# Painful Awakening due to *Scleroderma* Stings

**DOI:** 10.4269/ajtmh.20-0188

**Published:** 2020-10

**Authors:** Loïc Simon, Pascal Delaunay, Pierre Marty

**Affiliations:** 1 Parasitologie-Mycologie, Centre Hospitalier Universitaire de Nice, Université Côte d’Azur, Nice, France;; 2 MIVEGEC, UMR IRD 224-CNRS 5290-Université de Montpellier, Montpellier, France;; 3 Inserm U1065, Centre Méditerranéen de Médecine Moléculaire, Nice, France

**Figure 1.**
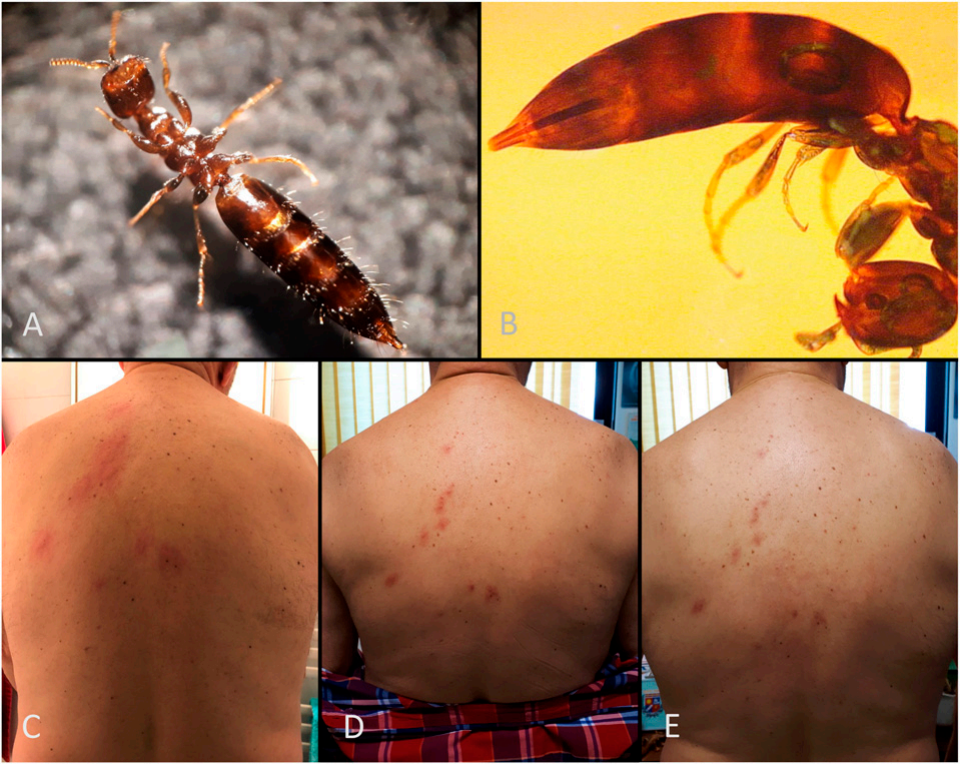
(**A**) *Scleroderma domesticum*, ventral view. (**B**) *S. domesticum* with ovipositor stinger. (**C**–**E**) Evolution of the papular lesions on the patient’s back (day 0, day 3, and day 7). This figure appears in color at www.ajtmh.org.

A 66-year-old male patient from Nice, French Riviera, suddenly woke up in the morning at his home feeling a severe sting-like pain, first in the neck and then in the back. He searched in his bed and found a 4-mm insect (Figure 1A and B). A pruritic rash with multiple inflammatory papular lesions in line was apparent (Figure 1C). The evolution was good without treatment. Inflammation disappeared in the next 2 hours. Three days later, the lesions were not painful anymore (Figure 1D). On day 7, healing lesions were still visible on the patient’s back (Figure 1E). The insect was identified as *Scleroderma domesticum*. Only the females are responsible for human lesions. They can be found near old wooden furniture where they parasitize xylophagous beetles larvae. They inject venom with their sting to paralyze the larvae and feed on their hemolymph before laying their eggs on them.^1^ This insect may be found in antique dealer stores because of the presence of its host.^2^ It very rarely causes human lesions. Its maximum activity is in the evening and at night, but daytime bites are possible. Eradicating furniture beetles is the key to prevent these exceptional human infestations.^3^
